# Short-Term Activation of Peroxisome Proliferator-Activated Receptors *α* and *γ* Induces Tissue-Specific Effects on Lipid Metabolism and Fatty Acid Composition in Male Wistar Rats

**DOI:** 10.1155/2019/8047627

**Published:** 2019-06-12

**Authors:** Elin Strand, Vegard Lysne, Mari Lausund Grinna, Pavol Bohov, Asbjørn Svardal, Ottar Nygård, Rolf K. Berge, Bodil Bjørndal

**Affiliations:** ^1^Department of Clinical Science, University of Bergen, Bergen, Norway; ^2^Department of Immunology and Transfusion Medicine, Haukeland University Hospital, Bergen, Norway; ^3^Centre for Nutrition, Department of Clinical Science, University of Bergen, Bergen, Norway; ^4^Department of Heart Disease, Haukeland University Hospital, Bergen, Norway; ^5^Mohn Nutrition Research Laboratory, Department of Clinical Science, University of Bergen, Bergen, Norway

## Abstract

Dietary fatty acids (FAs) affect certain metabolic routes, including pathways controlled by the peroxisome proliferator-activated receptors (PPARs), but tissue-specific effects are not well-defined. Thus, the aim was to compare the metabolic response in hepatic, adipose, and cardiac tissues after treatment with specific PPAR agonists. Male Wistar rats were randomized into three groups: a control group receiving placebo (n=8); a PPAR*α* agonist group receiving WY-14,643 (n=6); and a PPAR*γ* agonist group receiving rosiglitazone (n=6) for 12 days. All animals received a low-fat standard chow diet and were given a daily dose of placebo or agonist orally. Lipids and FA methyl esters were measured in plasma, liver, and heart and gene expression was measured in liver and adipose tissue, while enzyme activities were measured in liver. Treatment with the PPAR*α* agonist was associated with higher liver mass relative to body weight (liver index), lower plasma, and hepatic total cholesterol, as well as lower plasma carnitine and acylcarnitines, compared with control. In heart, PPAR*α* activation leads to overall lower levels of free FAs and specific changes in certain FAs, compared with control. Furthermore, *β*-oxidation in liver and the enzymatic activities of well-known PPAR*α* targeted genes were higher following PPAR*α* administration. Overall, rats treated with the PPAR*α* agonist had higher hepatic saturated FAs (SFAs) and monounsaturated FAs (MUFAs) and lower n-6 and n-3 PUFAs, compared to control. Treatment with the PPAR*γ* agonist was associated with a lower liver index, lower plasma triglycerides (TAG) and phospholipids, and higher hepatic phospholipids, compared with control. PPAR*γ* target genes were increased specifically in adipose tissue. Moreover, lower total cardiac FAs and SFA and higher cardiac n-6 PUFA were also associated with PPAR*γ* activation. Altogether, there were characteristic effects of PPAR*α* activation in liver and heart, as well as in plasma. PPAR*γ* effects were not only confined to adipose tissue, but specific effects were also seen in liver, heart, and plasma. In conclusion, short-term treatment with PPAR agonists induced tissue-specific effects on FA composition in liver and heart. Moreover, both PPAR*α* and PPAR*γ* activation lowered plasma TAG and phospholipids, most likely through effects on liver and adipose tissue, respectively. In future studies we aim to reveal whether similar patterns can be found through diet-induced activation of specific pathways.

## 1. Introduction

Peroxisome proliferator-activated receptors (PPARs) belong to the superfamily of nuclear receptors and are central transcription factors governing pathways involved in energy metabolism and homeostasis [1]. PPAR*α* is highly expressed in metabolically active tissues like the liver, heart, and kidneys and is involved in regulating genes related to glucose and lipid metabolism [2], while PPAR*γ* is abundantly expressed in adipose tissue, where it controls lipid storage and differentiation of adipocytes [3]. PPAR*γ* is also expressed in several other tissues, and its action includes traits like improved insulin sensitivity [4] and holds anti-inflammatory properties [5].

Since PPAR*α* has hypolipidemic and PPAR*γ* has insulin sensitizing properties, these transcription factors have been of large clinical interest as targets for medical treatment of conditions involving cardiovascular disease and diabetes [6, 7]. Fatty acids (FAs) and their derivatives are natural ligands for the PPARs, and diet will consequently have a significant impact on their activities [8]. An increased focus on dietary fat, including the proposed beneficial effects of polyunsaturated FAs [9] and trend diets like the high fat-low carbohydrate diet [10], necessitates studies on how the internal organs are affected. Well-known synthetic PPAR ligands include fibrates activating PPAR*α* and thiazolidinediones activating PPAR*γ* [1]. Although the main metabolic effects of PPAR*α* and *γ* have been documented in rodents and humans, few studies have performed such comprehensive and direct comparison of PPAR activation effects on lipid metabolism and lipid status across important metabolic organs.

The objective of the current study was to compare how short-term activation of PPAR*α* and **γ** affected metabolism through lipid levels and FA composition, as well as gene regulation in liver and adipose tissue. For this purpose, male Wistar rats were treated for 12 days with the specific PPAR agonists WY-14,643 (PPAR*α*) and rosiglitazone (PPAR*γ*), followed by measuring hepatic and cardiac lipids, FA composition, as well as certain hepatic and adipose genes expected to respond to PPAR activation.

## 2. Methods

### 2.1. Animals and Study Design

The animal experiments were standardized according to the Guidelines for the Care and Use of Experimental Animals, and the protocol was approved by the Norwegian Committee for Experiments on Animals, and in accordance with the Norwegian legislation and regulations governing experiments using live animals (FOTS ID: 2014/6187). The experiment was done in accordance with the regulations laid down by the National Animal Research Authority.

A total of 20 male Wistar rats, aged eight weeks (200-225 g), were obtained from Taconic Europe A/S (Ry, Denmark). They were housed 2-3 animals per cage (Makrolon IV). The animal room maintained a constant 12 h light–dark cycle at a temperature of 22 ± 2°C, a relative humidity of 55 ± 5%, and underwent 20 air changes per hour. Common environmental enrichment was used. Animals were acclimatized under these conditions for six days prior to study start and had free access to standard chow and tap water throughout the study. During the following two days prior to study intervention, animals were habituated careful handling, and they were introduced to the muffin dough to be used as a vehicle for the PPAR agonists.

The animals were randomized into the following three groups: (1) Placebo (control, n=8); (2) PPAR*α* agonist (n=6); and (3) PPAR*γ* agonist (n=6). Block randomization was used when placing the animals into cages, as well as for the terminal operation. Sample size in each group was determined based on an assumed profound response (if any) in the PPAR intervention groups. All animals received a low-fat chow diet. In addition, during the 12 days of study intervention each animal was given a daily supplement of 300 *μ*l muffin dough as a vehicle with or without agonist. Treatments were given according to the following daily routine: the placebo control group received pure vehicle (without agonist); the PPAR*α* agonist group received vehicle with 20 mg/kg/day WY-14,643 (Tocris Bioscience, Bristol, UK); and the PPAR*γ* agonist group received vehicle with 10 mg/kg/day rosiglitazone (Sigma-Aldrich, St. Louis, MO). Ingredients in the muffin dough vehicle were eggs, sugar, gluten free flour, vanilla sugar, milk, and butter. Animal weights were measured at day 0, day 6, and day 12 during the experiment. Feed intake was estimated by weighing the food provided to each cage and finally weighing the residual feed on day 12.

The animals received anesthesia with 2% isoflurane (Schering-Plough, Kent, UK) under fasting conditions after 12 days of intervention. The abdomen was opened in the midline and the animals were sacrificed by cardiac puncture and exsanguination. Blood was collected in BD Vacutainer tubes containing EDTA (Becton, Dickinson, and Company, Plymouth, UK). Liver, heart, and epididymal adipose tissue were collected, weighed, and snap-frozen as drainage of blood from the animal was complete. Liver mass relative to body weight (liver index) was calculated by the formula [100*∗*(liver weight in g/body weight in g)]. A liver piece for the *β*-oxidation was collected by cutting a piece of the main lobe. A small piece was also collected for FA analyses. Plasma and tissue samples were stored at −80°C until analyses.

### 2.2. Biochemical Analyses

Plasma, hepatic, and cardiac lipids were measured on the Hitachi 917 system (Roche Diagnostics, GmbH, Mannheim, Germany). Total cholesterol, HDL cholesterol, LDL cholesterol, and TAG kits were from Roche Diagnostics and the phospholipids, free cholesterol, and free FAs kits from DiaSys Diagnostic Systems GmbH (Holzheim, Germany). Plasma carnitine metabolites were determined by high performance liquid chromatography-tandem mass spectrometry (LC-MS/MS) as previously described [11–13]. FA methyl esters (FAME) were prepared from hepatic and cardiac tissues and analyzed by gas-liquid chromatography (GC) as previously described [14]. The anti-inflammatory index was calculated based on the formula ((C22:6n-3 + C22:5n-3 + C20:3n-6 + C20:5n-3) / C20:4n-6)*∗*10 [15].

### 2.3. Hepatic Enzyme Activities

Liver tissue samples were homogenized and fractionated as previously described [16]. The postnuclear fraction was used for further analyses. Liquid scintillation with (1-^14^C) palmitoyl-CoA as a substrate was used to determine *β*-oxidation capacity in liver in the absence and presence of malonyl-CoA [17]. The activities of carnitine palmitoyl transferase 2 (CPT-II) [18], fatty acyl-CoA oxidase (ACOX) [19, 20], and 3-hydroxy-3-methylglutaryl-CoA (HMG-CoA) synthase [21] were measured as previously described.

### 2.4. Gene Expression Analyses

Total cellular RNA was purified from tissue using the RNeasy kits with the RNeasy® Mini protocol for liver and the RNeasy® Lipid Tissue protocol for adipose tissue (Qiagen GmbH, Hilden, Germany). RNA quantity was determined spectrophotometrically (NanoDrop 1000, Thermo Scientific, Wilmington, DE, USA), while quality was evaluated by capillary electrophoresis (Agilent 2100 Bioanalyzer, Agilent Technologies Inc., Santa Clara, CA, USA). RNA was reversely transcribed to cDNA in 20 *μ*l reactions (containing 500 mg RNA) using the High Capacity cDNA Reverse Transcription Kit (Applied Biosystems, Foster City, CA, USA). Samples were treated with RNase inhibitors as part of the protocol. Selected genes were analyzed using qPCR with the ABI PRISM 7900 HT Sequence Detection System (Applied Biosystems): Rn00566193 (*Pparα*), Rn00440945 (*Pparɣ*), Rn00580241 (*Pgc1α*), Rn00580702 (*Cpt1a*), Rn00563995 (*Cpt2*), Rn00571166 (*Ucp2*), Rn01460628 (*Acox1*), Rn00597339 (*Hmgcs2*), Rn00569117 (*Fas*), Rn00580728 (*Cd36*), Rn00664587 (*Fabp1*), Rn04219585 (*Fabp4*), Rn00585821 (*Fatp1*), Rn00561482 (*Lpl*), Rn01423343 (*Pltp*), Rn00561474 (*Lipc*), Rn00563444 (*Lipe*), Rn00562483 (*ApoA1*), Rn01499050 (*ApoB*), Rn01764530 (*ApoC2*), and Rn00560743 (*ApoC3*). All primer/probe sequences for the genes studied were obtained from Applied Biosystems. The MIQE guidelines for qPCR analyses were used when selecting house-keeping genes [22, 23]. Three house-keeping genes were tested: RT-CKFT-18s (*18 S*, Eurogentec S.A., Seraing, Belgium), Rn03302271_gH (*Rplp0*, Applied Biosystems), and Rn00821091_g1 (*Rplp4*, Applied Biosystems). The house-keeping gene* Rplp4* was found to be the best using NormFinder [24] and was used to normalize the expression value of each gene in all samples.

### 2.5. Statistical Analyses

All measurements except the gene expression data were log-transformed and presented as geometric means with their geometric standard deviations (gSD). Gene expression data was normalized against the control group (placebo) and presented as mean (SD) relative to the control group. The groups were compared by a one-way ANOVA, and the proportion of variance explained by the experimental groups was assessed by calculating the *η* ^2^. The assumption of equal variance was assessed with Levene's test and visually by plotting the residuals. Within-group normality was assessed visually by Q-Q plots of the residuals. Planned comparisons towards the control group were performed for the two intervention groups, and p-values were extracted from the regression model. Standardized mean difference (SMD; 95% confidence interval) were calculated and plotted to illustrate the differences from the control group. The p-values were adjusted using the method of Benjamini and Hochberg [25] to control the false discovery rate. The raw individual values were plotted with overlaying box plots. The data file was processed in IBM SPSS Statistics for Windows, version 23 (IBM Corporation, Armonk, NY, USA), and statistics were performed in R version 3.5.1 (https://www.R-project.org/), and the packages within the tidyverse (*dplyr*,* broom*,* purr*,* magrittr*, and* rlang*) and forestplot. P-values <0.05 were considered as statistically significant.

## 3. Results

### 3.1. Weights and Lipid Related Parameters

**Figure 1:**
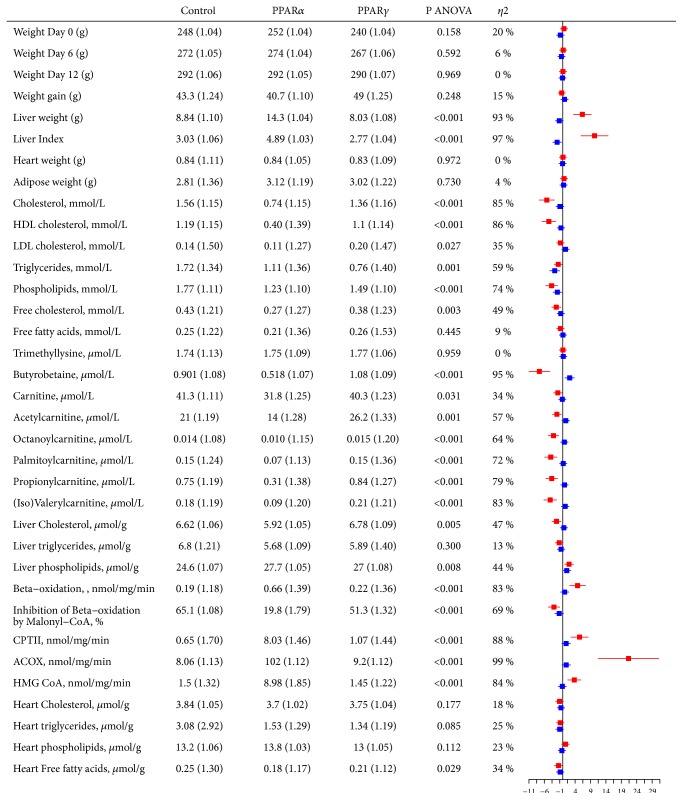
Weights and lipid related parameters in Wistar rats during treatment with PPAR agonists for 12 days. Values are geometric mean (gSD). Red bars correspond to PPAR*α* vs control and blue bars to PPAR*γ* vs control. Measurements of variables are in plasma, unless otherwise stated. ACOX, fatty acyl-CoA oxidase; ANOVA, analysis of variance; CPTII, carnitine palmitoyltransferase II; EPI, epididymal fat; HDL, high density lipoprotein; HMG-CoA, 3-hydroxy-3-methylglutaryl-coenzyme A synthase; LDL, low density lipoprotein; PPAR, peroxisome proliferator-activated receptor.

Details on weight measurements and lipid related parameters are illustrated in Figure 1. At study start geometric mean (gSD) weight of the animals was 247 (1.04) gram and during 12 days they gained 44.0 (1.22) gram. Neither baseline weights nor weight gain significantly differed between treatment groups. Of interest, the weights of the epididymal adipose tissue did not differ between groups treated with agonists, compared to control after 12 days intervention.

Treatment with WY-14,643 was associated with higher liver weight and liver index (SMD = 6.4 and 10.3), lower plasma total and HDL cholesterol (SMD = -5.2 and -4.5), triglycerides (TAG; SMD = -1.5), phospholipids (SMD = -3.6), and free cholesterol (SMD = -2.1), compared with control. Furthermore, animals receiving the PPAR*α* agonist had lower plasma levels of the carnitine precursor butyrobetaine (SMD = -7.6), as well as carnitine (SMD = -1.6) and all measured acylcarnitines (SMD = -4.1 - -1.9).

In liver, PPAR*α* activation was associated with lower total cholesterol (SMD = -2.0) and higher phospholipids (SMD = 2.0). *β*-oxidation was higher (SMD = 4.8), while the ability of inhibition by malonyl-CoA was substantially reduced (SMD = -2.9). The activities of carnitine palmitoyltransferase II (CPTII) (SMD = 5.4), fatty acyl-CoA oxidase (ACOX; SMD = 21.3), and 3-hydroxy-3-methylglutaryl-coenzyme A (HMG-CoA) synthase (SMD = 3.7) were all higher following treatment with WY-14,643.

Free cardiac FAs were lower following PPAR*α* activation, compared with control (SMD = -1.4).

Treatment with rosiglitazone was associated with a lower liver index (SMD = -1.8), lower plasma TAG (SMD = -2.6) and phospholipids (SMD = -1.7), higher plasma butyrobetaine (SMD = 2.2), and higher hepatic phospholipids (SMD = 1.3).

Supplemental Figure 1 illustrates the raw values of each measured parameter from Figure 1.

Feed intake was estimated throughout the study, and the rats who received the PPAR*γ* agonist had a significantly higher intake of chow compared with control (*P*<0.001). However, there was no statistically significant impact on weight gain or feed efficiency (weight gain per feed intake (g)) in either of the groups.

### 3.2. Fatty Acid Composition in Liver and Heart

**Figure 2:**
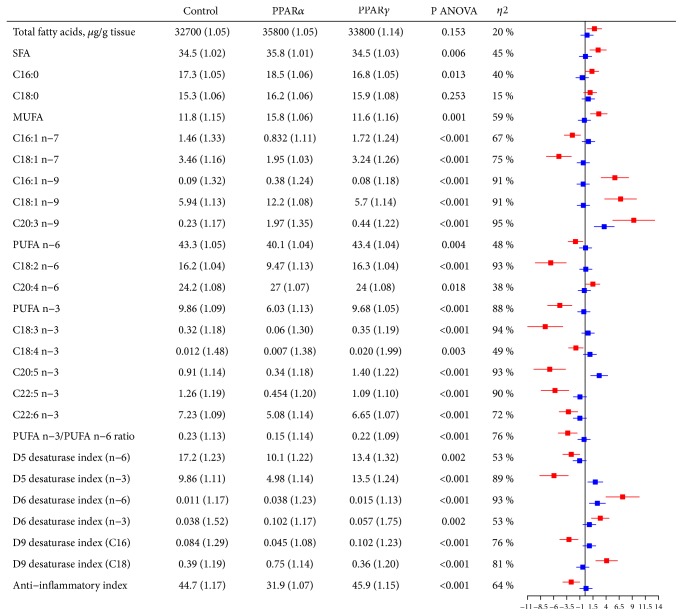
Hepatic fatty acid composition (wt%) in Wistar rats after treatment with PPAR agonists for 12 days. Values are geometric mean (gSD). Red bars correspond to PPAR*α* vs control and blue bars to PPAR*γ* vs control. ANOVA, analysis of variance; MUFA, monounsaturated fatty acids; PPAR, peroxisome proliferator-activated receptor; PUFA, polyunsaturated fatty acids; SFA, saturated fatty acids. D5 desaturase index (n-6) = C20:4n-6 / C20:3n-6 (an indirect index of Δ5 desaturase activity based on n-6 PUFA). D5 desaturase index (n-3) = C20:5n-3 / C20:4n-3 (an indirect index of Δ5 desaturase activity based on n-3 PUFA). D6 desaturase index (n-6) = C18:3n-6 / C18:2n-6 (an indirect index of Δ6 desaturase activity based on n-6 PUFA). D6 desaturase index (n-3) = C18:4n-3 / C18:3n-3 (an indirect index of Δ6 desaturase activity based on n-3 PUFA). D9 desaturase index (C16) = C16:1n-7 / C16:0 (an indirect index of Δ9 desaturase activity based on C16 SFA/MUFA). D9 desaturase index (C18) = C18:1n-9 / C18:0 (an indirect index of Δ9 desaturase activity based on C18 SFA/MUFA). Anti-inflammatory index = ((C22:6n-3 + C22:5n-3 + C20:3n-6 + C20:5n-3) / C20:4n-6)*∗*100.

Overall, rats treated with the PPAR*α* agonist had higher hepatic SFA (SMD = 2.5) and MUFA (SMD = 2.6) and lower n-6 (SMD = -1.8) and n-3 (SMD = -4.8) PUFAs, compared to control (Figure 2). Along the MUFA pathway there was a downstream increase towards a final higher mead acid (C20:3n-9; SMD = 9.3). PPAR*α* activation was associated with lower hepatic content of the essential PUFAs linoleic (C18:2n-6) and *α*-linolenic (C18:3n-3) acids (SMD = -6.6 and -7.6), but with higher arachidonic acid (C20:4n-6; SMD = 1.5) and lower levels of the most downstream n-3 PUFAs (C20-22; SMD = -6.8, -5.8, and -3.2). Altogether, this was in line with a lower PUFA n-3/n-6 ratio (SMD = -3.3) and anti-inflammatory index (SMD = -2.6) after WY-14,643 treatment. Furthermore, there was an overall lower estimated Δ5 desaturase activity and a higher estimated Δ6 desaturase activity in animals treated with the PPAR*α* agonist. The Δ9 desaturase index based on C16 was lower (SMD = -3.1), while that based on C18 was higher (SMD = 4.1).

The strongest effects on hepatic FA composition following treatment with rosiglitazone were higher contents of mead acid (C20:3n-9; SMD = 3.6) and eicosapentaenoic acid (C20:5n-3; SMD = 2.7) compared to control, the last mentioned also illustrated by the increase in estimated Δ5 desaturase activity along the n-3 pathway (SMD = 1.9).

Supplemental Figure 2 illustrates the raw values of each measured parameter from Figure 2. FA composition in plasma was very similar to that observed in hepatic tissue, with few exceptions (Supplemental Figure 3).

**Figure 3:**
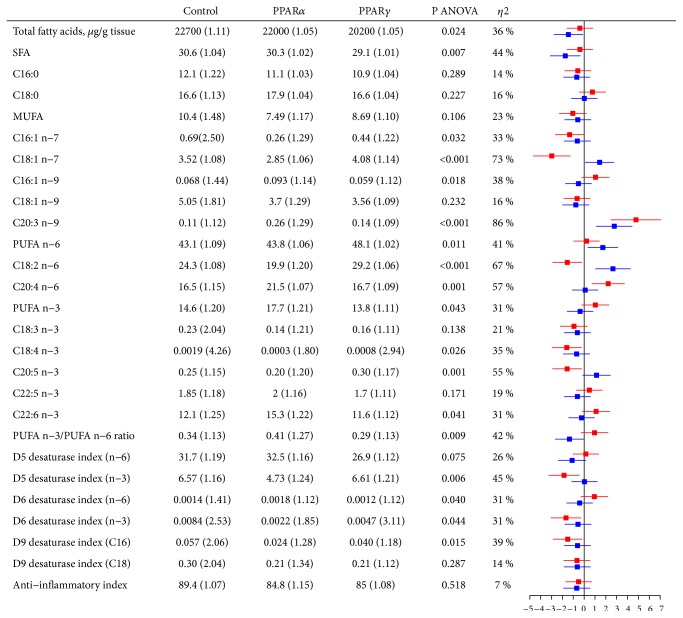
Cardiac fatty acid composition (wt%) in Wistar rats after treatment with PPAR agonists for 12 days. Values are geometric mean (gSD). Red bars correspond to PPAR*α* vs control and blue bars to PPAR*γ* vs control. ANOVA, analysis of variance; MUFA, monounsaturated fatty acids; PPAR, peroxisome proliferator-activated receptor; PUFA, polyunsaturated fatty acids; SFA, saturated fatty acids. D5 desaturase index (n-6) = C20:4n-6 / C20:3n-6 (an indirect index of Δ5 desaturase activity based on n-6 PUFA). D5 desaturase index (n-3) = C20:5n-3 / C20:4n-3 (an indirect index of Δ5 desaturase activity based on n-3 PUFA). D6 desaturase index (n-6) = C18:3n-6 / C18:2n-6 (an indirect index of Δ6 desaturase activity based on n-6 PUFA). D6 desaturase index (n-3) = C18:4n-3 / C18:3n-3 (an indirect index of Δ6 desaturase activity based on n-3 PUFA). D9 desaturase index (C16) = C16:1n-7 / C16:0 (an indirect index of Δ9 desaturase activity based on C16 SFA/MUFA). D9 desaturase index (C18) = C18:1n-9 / C18:0 (an indirect index of Δ9 desaturase activity based on C18 SFA/MUFA). Anti-inflammatory index = ((C22:6n-3 + C22:5n-3 + C20:3n-6 + C20:5n-3) / C20:4n-6)*∗*100.

Certain changes were also seen in heart tissue following treatment with the PPAR agonists (Figure 3). Administration of WY-14,643 was associated with higher mead acid (C20:3n-9; SMD = 4.8), lower linoleic acid (C18:2n-6; SMD = -1.5), and higher arachidonic acid (C20:4n-6; SMD = 2.2). Treatment with rosiglitazone was associated with lower total cardiac FAs (SMD = -1.5) and SFA (SMD = -1.7) and higher cardiac n-6 PUFA (SMD = 1.7) due to higher linoleic acid (C18:2n-6; SMD = 2.7), compared to control.

Supplemental Figure 4 illustrates the raw values of each measured parameter from Figure 3.

### 3.3. Gene Expression in Liver and Epididymal Adipose Tissue

**Figure 4:**
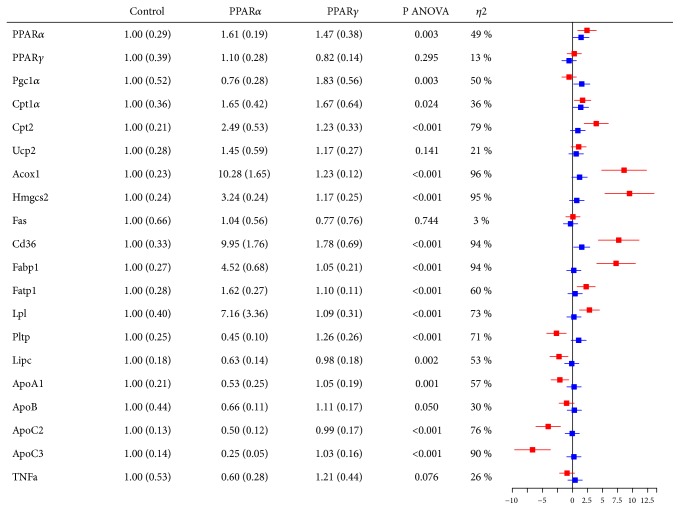
Hepatic gene expression in Wistar rats after treatment with PPAR agonists for 12 days, normalized towards the control group. Red bars correspond to PPAR*α* vs control and blue bars to PPAR*γ* vs control. Acox1, fatty acyl-CoA oxidase 1; ANOVA, analysis of variance; ApoA1, apolipoprotein A1; ApoB, apolipoprotein B; ApoC2, apolipoprotein C2; ApoC3, apolipoprotein C3; Cd36, cluster of differentiation 36; Cpt1a, carnitine palmitoyltransferase 1a; Cpt2, carnitine palmitoyltransferase 2; Fabp1, fatty acid binding protein 1; Fas, fatty acid synthase; Fatp1, fatty acid transport protein 1; Hmgcs2, 3-hydroxy-3-methylglutaryl-CoA synthase 2; Lipc, hepatic lipase C; Lpl, lipoprotein lipase; Pgc1*α*, peroxisome proliferator-activated receptor ɣ coactivator 1*α*; Pltp, phospholipid transfer protein; Ppar*α*, peroxisome proliferator-activated receptor *α*; Pparɣ, peroxisome proliferator-activated receptor ɣ; TNFa, tumor necrosis factor alpha; Ucp2, uncoupling protein 2.

Several established PPAR target genes were affected in liver after treatment with WY-14,643 (Figure 4). Fatty acyl-CoA oxidase 1 (*Acox1*) is the rate-limiting enzyme of peroxisomal *β*-oxidation, whereas carnitine palmitoyltransferases 1a and 2 (*Cpt1a and Cpt2*) are involved in the transfer of FAs into the mitochondria. Under certain metabolic circumstances acetyl-CoA is broken down by the rate-limiting enzyme 3-hydroxy-3-methylglutaryl-CoA synthase 2 (*Hmgcs2*) into ketone bodies. Transport proteins important for FAs include Cluster of differentiation 36 (*Cd36*), fatty acid binding protein 1 (*Fabp1*), and fatty acid transport protein 1 (*Fatp1*). Lipoprotein lipase (*Lpl*) is activated by Apolipoprotein C2 (*ApoC2*) and contributes to increased lipolysis of TAG. Whereas* PPARα*,* Cpt1a*,* Cpt2*,* Acox1*,* Hmgcs2*,* Cd36*,* Fabp1*,* Fatp1*, and* Lpl *were upregulated (SMD = 1.7 – 9.5),* Pltp*,* Lipc*,* ApoA1*,* ApoC2*, and* ApoC3* (SMD = -6.6 - -2.1) were downregulated, compared with control. Only few hepatic genes were appreciably affected by treatment with rosiglitazone. These were* PPARα*,* Pgc1α*,* Cpt1a*, and* Cd36*, which were all upregulated (SMD = 1.4 – 1.5).

Supplemental Figure 5 illustrates the raw values of gene expression relative to control from Figure 4.

**Figure 5:**
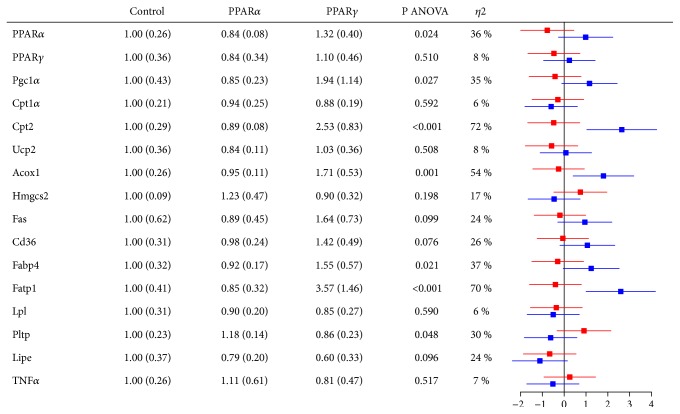
Epididymal adipose tissue gene expression in Wistar rats after treatment with PPAR agonists for 12 days, normalized towards the control group. Red bars correspond to PPAR*α* vs control and blue bars to PPAR*γ* vs control. Acox1, fatty acyl-CoA oxidase 1; ANOVA, analysis of variance; Cd36, cluster of differentiation 36; Cpt1a, carnitine palmitoyltransferase 1a; Cpt2, carnitine palmitoyltransferase 2; Fabp4, fatty acid binding protein 4; Fas, fatty acid synthase; Fatp1, fatty acid transport protein 1; Hmgcs2, 3-hydroxy-3-methylglutaryl-CoA synthase 2; Lipe, hormone sensitive lipase E; Lpl, lipoprotein lipase; Pgc1*α*, peroxisome proliferator-activated receptor ɣ coactivator 1*α*; Pltp, phospholipid transfer protein; Ppar*α*, peroxisome proliferator-activated receptor *α*; Pparɣ, peroxisome proliferator-activated receptor ɣ; TNFa, tumor necrosis factor alpha; Ucp2, Uncoupling protein 2.

In epididymal adipose tissue, no genes were clearly affected by PPAR*α* administration, whereas* Cpt2*,* Acox1*,* Fabp4*, and* Fatp1* were upregulated by PPAR*γ* administration (SMD = 1.2 - 2.6) (Figure 5).

Supplemental Figure 6 illustrates the raw values of gene expression relative to control from Figure 5.

## 4. Discussion

**Figure 6:**
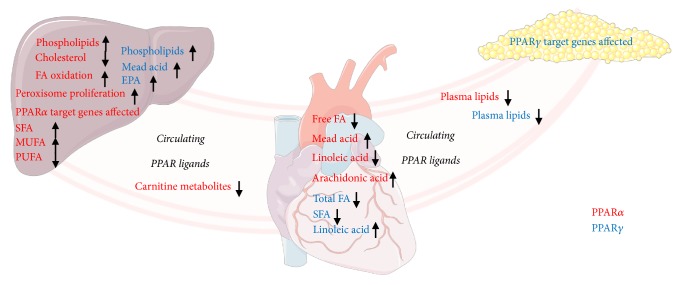
A summary showing main effects of PPAR*α* and PPAR*γ* activation as observed in the current study in the circulation, liver, heart, and adipose tissue as indicated. PPAR*α* effects are shown in red, while PPAR*γ* effects are shown in blue.

Overall, in the current short-term study of PPAR*α* and PPAR*γ* activation, we present a comprehensive report with specific effects on circulating lipids, FA composition in liver, heart, and plasma and expression of known PPAR target genes in liver and adipose tissue. PPAR*α* activation was obtained by treatment with WY-14,643, while PPAR*γ* activation was obtained by treatment with rosiglitazone. PPAR*α* activation was associated with lower plasma total cholesterol, HDL cholesterol, TAG, and phospholipids, higher hepatic phospholipids, SFA and MUFA, lower hepatic n-6 and n-3 PUFA, and higher cardiac arachidonic acid. PPAR*γ* activation was associated with lower plasma TAG and phospholipids, lower cardiac total FAs and SFA, and higher cardiac n-6 PUFA. A summary of characteristic effects of PPAR*α* and PPAR*γ* activation in the circulation, as well as in liver, heart, and adipose tissue is illustrated in Figure 6.

The effects of PPAR*α* agonist stimulation on hepatic lipid metabolism in rodents are well-documented and include induced FA uptake, *β*-oxidation, ketogenesis, and TAG clearance [26]. Similar effects are observed in heart and skeletal muscle, but to a much lesser degree due to the tissue-specific expression pattern of PPAR*α* [27]. Thus, liver plays a major role in PPAR*α*-induced plasma lipid lowering. Moreover, hepatic peroxisome proliferation, related to increased liver weight, is a well-known effect in response to PPAR stimulation in rodents [28]. In addition, activation of PPAR*α* will result in body weight loss after prolonged treatment [29, 30]. PPAR*γ* agonists, in contrast, have more prominent effects on adipose tissue. Rosiglitazone has been shown to stimulate the lipid storage capacity of adipose tissue, increase lipid uptake, and efficiently reduce plasma glucose levels [31–33]. Activation of PPAR*γ* has been postulated to regulate the expression of adipose triglyceride lipase (ATGL), which has an important role in lipid metabolism [34]. Furthermore, rosiglitazone treatment has been associated with increased ATGL expression in WAT and BAT of lean and obese mice [34].

Although the roles of PPAR*α* and -*γ* on lipid metabolism in rodents have been described previously, few studies have performed a comprehensive comparison of PPAR*α* and PPAR*γ* agonists on lipids, FA composition, and gene expression in major organs involved in lipid turnover. Findings in animals treated with the PPAR*α* agonist indicated an increased hepatic *β*-oxidation and ketogenesis compared to rats treated with vehicle. An enhanced FA transport and uptake is supported by an increased hepatic expression of* Cd36*,* Fabp1*, and* Fatp1* [26]. Interestingly, gene expression in adipose tissue was not influenced by two weeks of WY-14,643 treatment. Rosiglitazone treatment led to higher expression of hepatic* Pgc1a*,* Cpt1a*, and* Cd36*, although not to an extent comparable with the effect of WY-14,643 treatment. Moreover, pharmacological activation of PPAR*γ* was associated with higher expression of adipose* Cpt2*,* Acox1*,* Fabp4, *and* Fatp1* compared with control. As there was a strong plasma TAG reducing effect after PPAR*γ* activation, our results indicate that PPAR*γ*-induced lipid transport and catabolism in adipose tissue may contribute to the reduction in plasma TAG levels. Similarly, short-term PPAR*γ* activation, through rosiglitazone therapy, showed reduced plasma TAG and NEFAs in Wistar rats [35]. Moreover, Harrington et al. showed that a PPAR*γ* agonist GW7845 reduced plasma TAG levels in AKR/J mice treated for four weeks, which did not relate to increased* in vitro* hepatic FA oxidation [36]. PPAR*γ* activation has previously shown to reduce plasma TAG in rats through adipose tissue-specific increase in LPL activity as well as increased gene expression of FA transport proteins, and this process requires mTOR activity [37, 38]. Increased LPL activity has been associated with enhanced lipolytic activity, which in turn is associated with TAG clearance [39]. In the current study, gene expression patterns demonstrated minor decrease in* Lpl* in adipose tissue following treatment with both PPAR agonists. On the contrary, there was an enhanced activity of genes associated with *β*-oxidation in adipose tissue following rosiglitazone treatment.

Several studies have shown that PPAR*γ* agonists increase feed intake and body weight and reduce plasma glucose and insulin levels, while the PPAR*α* agonists reduce body weight [36]. We did not observe any statistically significant difference in weight gain between the treatment groups, despite an increase in feed intake among PPAR*γ* supplemented rats. Moreover, glucose levels were unchanged. This could be due to the relatively short treatment period, as the PPAR*γ* group did gain more weight in absolute terms.

Plasma carnitine metabolites were in general reduced after treatment with WY-14,643, but there were minor effects after treatment with rosiglitazone. Treatment of rats with a PPAR*α* specific agonist [40] and a pan PPAR agonist with main affinity for PPAR*α* [41] did also demonstrate reduced plasma acylcarnitine levels and increased expression of the* Bbox1 *gene involved in production of the carnitine precursor butyrobetaine. Carnitine is essential for the transport of medium and long fatty acyl chains in and out of the mitochondrion, and plasma levels may reflect intracellular levels. Thus, the reduction in plasma carnitine and its precursor butyrobetaine by PPAR*α* may have been due to an increased utilization of carnitine for FA transport, while the reduction in acylcarnitines could be linked to the increased *β*-oxidation and ketogenesis, lowering the levels of intermediate and end-products of *β*-oxidation. In line with this, acylcarnitines have been proposed as sensors of mitochondrial FA oxidation [42], and high levels of palmitoylcarnitine and octanoylcarnitine are linked to poor prognosis in patients with cardiovascular disease [13, 43]. Our results indicate that PPAR*γ* has little influence on plasma acylcarnitines, despite the increased expression of genes involved in adipose tissue *β*-oxidation.

Hepatic peroxisome proliferation leads to induced peroxisomal- and as an indirect consequence also mitochondrial *β*-oxidation [44]. This strong effect on liver exerted by PPAR*α* may be reflected by its impact on hepatic FA composition. Although peroxisome proliferation does not occur in a similar manner in humans as in rodents, changes in FA composition in relation to *β*-oxidation rate can provide clues in the search for possible FA markers indicating PPAR activation under certain circumstances also in humans. It is conceivable that an increased peroxisomal catabolism of very long-chain FAs and subsequent oxidation in the mitochondria may induce shifts in FA composition. A study in ageing rats indicated that extent of peroxisomal *β*-oxidation affected brain FA composition [44]. Moreover, WY-14643 affected FA composition of myocardial phospholipids [45]. We have previously studied long-term PPAR*α* activation in rats, resulting in elevated cardiac levels, as opposed to lower hepatic levels of n-3 PUFAs [14]. These tissue-specific differences are similar to those observed in the current short-term study, including lower hepatic n-3 PUFA and a tendency of elevated cardiac n-3 PUFA levels. The lower hepatic level of n-3 PUFAs in animals treated with PPAR*α* agonist may be a consequence of the increased hepatic *β*-oxidation, particularly since the long-chain n-3 PUFAs are preferred FA substrates for *β*-oxidation [46]. Interestingly, in both studies, mead acid was elevated in liver and heart, a PPAR mechanism which may be linked with an essential FA deficiency [47]. It is not straight-forward to interpret exactly which mechanisms can be related to levels of SFAs and MUFAs, as these are more dependent on endogenous conversion compared to those of the PUFA subtype which are more directly related to the dietary intake [48].

In the current study, focus has been on PPAR*α*- and *γ* specific effects in metabolic active tissues, including liver, heart, and adipose tissue. Altogether, induced *β*-oxidation and enzymatic activity of PPAR*α* target proteins were supportive of well-known PPAR*α* specific effects in liver. Short-term treatment with rosiglitazone, a PPAR*γ* specific synthetic agonist, reduced plasma TAG in male Wistar rats, which may relate to adipose tissue-specific effects on mitochondrial function and lipid uptake. Moreover, PPAR*γ* activity seemed to affect *β*-oxidation in adipose tissue. Altogether, the current study demonstrates that PPAR*α* and PPAR*γ* specific ligands will influence lipids, gene expression, as well as FA composition in a tissue-specific manner. These findings are important for future studies on dietary components, when investigating which traits can be associated with PPAR related effects. It is also interesting to reveal PPAR effects on carnitine metabolites, which are potential biomarkers in human disease [49].

## 5. Conclusions

Short-term treatment with synthetic PPAR*α* and PPAR*γ* agonists induced changes in circulating lipids and FA composition in liver and heart, modifying mitochondrial function in a tissue-specific manner. Interestingly, we observed TAG and phospholipid lowering effects in plasma after treatment with both agonists. The ultimate future aim is to gain knowledge on how these parameters may be affected not only by activation through specific PPAR agonists, but also by dietary FAs as well as other dietary and lifestyle related factors. More knowledge regarding PPAR action may be a part of the puzzle when laying the foundation for patient-specific dietary and medical prevention and treatment of metabolic diseases like obesity, diabetes, and cardiovascular diseases.

## Data Availability

The SPSS data used to support the findings of this study are available from the corresponding author upon request.

## Supplementary Materials

Supplementary Materials Supplemental Figure 1: individual data on weights and lipid related parameters in Wistar rats during treatment with PPAR agonists for 12 days. Supplemental Figure 2: individual data on hepatic fatty acid composition (wt%) in Wistar rats after treatment with PPAR agonists for 12 days. Supplemental Figure 3: plasma fatty acid composition (wt%) illustrated as (A) lines indicating geometric mean (gSD) values and as (B) individual raw data on plasma fatty acid composition (wt%) in Wistar rats after treatment with PPAR agonists for 12 days. Red bars correspond to PPAR*α* vs control and blue bars to PPAR*γ* vs control. ANOVA, analysis of variance; MUFA, monounsaturated fatty acids; PPAR, peroxisome proliferator-activated receptor; PUFA, polyunsaturated fatty acids; SFA, saturated fatty acids. Supplemental Figure 4: individual data on cardiac fatty acid composition (wt%) in Wistar rats after treatment with PPAR agonists for 12 days. Supplemental Figure 5: individual hepatic gene expression, normalized towards the control group. Supplemental Figure 6: individual epididymal adipose tissue gene expression, normalized towards the control group.

## References

[1] Grygiel-Górniak B. (2014). Peroxisome proliferator-activated receptors and their ligands: nutritional and clinical implications—a review. *Nutrition Journal *.

[2] Duval C., Muller M., Kersten S. (2007). ARalpha-ARalphand dyslipidemia. *Biochimica et Biophysica Acta*.

[3] Rosen E. D., Walkey C. J., Puigserver P., Spiegelman B. M. (2000). Transcriptional regulation of adipogenesis. *Genes & Development*.

[4] Salvadó L., Serrano-Marco L., Barroso E., Palomer X., Vázquez-Carrera M. (2012). Targeting PPAR*β*/*δ* for the treatment of type 2 diabetes mellitus. *Expert Opinion on Therapeutic Targets*.

[5] Szanto A., Nagy L. (2008). The many faces of PPAR*γ*: anti-inflammatory by any means?. *Immunobiology*.

[6] Fruchart J. C., Staels B., Duriez P. (2001). The role of fibric acids in atherosclerosis. *Current Atherosclerosis Reports*.

[7] Sahajpal N. S., Jain S. K. (2016). Molecular remodeling of the insulin receptor pathway by thiazolidinediones in type 2 diabetes mellitus: A brief review. *Protein and Peptide Letters*.

[8] Krey G., Braissant O., L'Horset F. (1997). Fatty acids, eicosanoids, and hypolipidemic agents identified as ligands of peroxisome proliferator-activated receptors by coactivator-dependent receptor ligand assay. *Molecular Endocrinology*.

[9] Zock P. L., Blom W. A. M., Nettleton J. A., Hornstra G. (2016). Progressing Insights into the Role of Dietary Fats in the Prevention of Cardiovascular Disease. *Current Cardiology Reports*.

[10] Estruch R., Martínez-González M. A., Corella D. (2016). Effect of a high-fat Mediterranean diet on bodyweight and waist circumference: a prespecified secondary outcomes analysis of the PREDIMED randomised controlled trial. *The Lancet Diabetes & Endocrinology*.

[11] Vernez L., Wenk M., Krähenbühl S. (2004). Determination of carnitine and acylcarnitines in plasma by high-performance liquid chromatography/electrospray ionization ion trap tandem mass spectrometry. *Rapid Communications in Mass Spectrometry*.

[12] Bjørndal B., Burri L., Wergedahl H., Svardal A., Bohov P., Berge R. K. (2012). Dietary supplementation of herring roe and milt enhances hepatic fatty acid catabolism in female mice transgenic for hTNF*α*. *European Journal of Nutrition*.

[13] Strand E., Pedersen E. R., Svingen G. F. T. (2017). Serum acylcarnitines and risk of cardiovascular death and acute myocardial infarction in patients with stable angina pectoris. *Journal of the American Heart Association*.

[14] Strand E., Bjorndal B., Nygard O. (2012). Long-term treatment with the pan-PPAR agonist tetradecylthioacetic acid or fish oil is associated with increased cardiac content of n-3 fatty acids in rat. *Lipids in Health and Disease*.

[15] Chavali S. R., Zhong W. W., Utsunomiya T., Forse R. A. (1997). Decreased production of interleukin-1-beta, prostaglandin-E2 and thromboxane-B2, and elevated levels of interleukin-6 and -10 are associated with increased survival during endotoxic shock in mice consuming diets enriched with sesame seed oil supplemented with Quil-A saponin. *International Archives of Allergy and Immunology*.

[16] Berge R. K., Flatmark T. (1984). Osmundsen H: Enhancement of long-chain acyl-CoA hydrolase activity in peroxisomes and mitochondria of rat liver by peroxisomal proliferators. *European Journal of Biochemistry*.

[17] Willumsen N., Hexeberg S., Skorve J., Lundquist M., Berge R. K. (1993). Docosahexaenoic acid shows no triglyceride-lowering effects but increases the peroxisomal fatty acid oxidation in liver of rats. *Journal of Lipid Research*.

[18] Madsen L., Froyland L., Dyroy E., Helland K., Berge R. K. (1998). Docosahexaenoic and eicosapentaenoic acids are differently metabolized in rat liver during mitochondria and peroxisome proliferation. *The Journal of Lipid Research*.

[19] Berge R. K., Flatmark T., Christiansen E. N. (1987). Effect of a high-fat diet with partially hydrogenated fish oil on long-chain fatty acid metabolizing enzymes in subcellular fractions of rat liver. *Archives of Biochemistry and Biophysics*.

[20] Berge R. K., Nilsson A., Husøy A.-M. (1988). Rapid stimulation of liver palmitoyl-CoA synthetase, carnitine palmitoyltransferase and glycerophosphate acyltransferase compared to peroxisomal *β*-oxidation and palmitoyl-CoA hydrolase in rats fed high-fat diets. *Biochimica et Biophysica Acta (BBA) - Lipids and Lipid Metabolism*.

[21] Clinkenbeard K. D., Reed W. D., Mooney R. A., Lane M. D. (1975). Intracellular localization of the 3-hydroxy-3-methylglutaryl coenzme A cycle enzymes in liver. Separate cytoplasmic and mitochondrial 3-hydroxy-3-methylglutaryl coenzyme A generating systems for cholesterogenesis and ketogenesis.. *The Journal of Biological Chemistry*.

[22] Bustin S. A., Benes V., Garson J. A. (2009). The MIQE guidelines: minimum information for publication of quantitative real-time PCR experiments. *Clinical Chemistry*.

[23] Nolan T., Hands R. E., Bustin S. A. (2006). Quantification of mRNA using real-time RT-PCR. *Nature Protocols*.

[24] Andersen C. L., Jensen J. L., Ørntoft T. F. (2004). Normalization of real-time quantitative reverse transcription-PCR data: a model-based variance estimation approach to identify genes suited for normalization, applied to bladder and colon cancer data sets. *Cancer Research*.

[25] Benjamini Y., Hochberg Y. (1995). Controlling the false discovery rate: a practical and powerful approach to multiple testing. *Journal of the Royal Statistical Society B: Methodological*.

[26] Burri L., Thoresen G. H., Berge R. K. (2010). The role of PPAR activation in liver and muscle. *PPAR Research*.

[27] Braissant O., Foufelle F., Scotto C., Dauça M., Wahli W. (1996). Differential expression of peroxisome proliferator-activated receptors (PPARs): tissue distribution of PPAR-*α*, -*β*, and -*γ* in the adult rat. *Endocrinology*.

[28] Dzhekova-Stojkova S., Bogdanska J., Stojkova Z. (2001). Peroxisome proliferators: their biological and toxicological effects. *Clinical Chemistry and Laboratory Medicine*.

[29] Perreault M., Will S., Panza D. (2010). Modulation of nutrient sensing nuclear hormone receptors promotes weight loss through appetite suppression in mice. *Diabetes, Obesity and Metabolism*.

[30] Rachid T. L., Penna-de-Carvalho A., Bringhenti I., Aguila M. B., Mandarim-de-Lacerda C. A., Souza-Mello V. (2015). PPAR-alpha agonist elicits metabolically active brown adipocytes and weight loss in diet-induced obese mice. *Cell Biochemistry & Function*.

[31] Festuccia W. T., Blanchard P.-G., Turcotte V. (2009). Depot-specific effects of the PPARgamma agonist rosiglitazone on adipose tissue glucose uptake and metabolism. *Journal of Lipid Research*.

[32] Laplante M., Sell H., MacNaul K. L., Richard D., Berger J. P., Deshaies Y. (2003). PPAR-gamma activation mediates adipose depot-specific effects on gene expression and lipoprotein lipase activity: mechanisms for modulation of postprandial lipemia and differential adipose accretion. *Diabetes*.

[33] Laplante M., Festuccia W. T., Soucy G. (2006). Mechanisms of the depot specificity of peroxisome proliferator-activated receptor *γ* action on adipose tissue metabolism. *Diabetes*.

[34] Kershaw E. E., Schupp M., Guan H.-P., Gardner N. P., Lazar M. A., Flier J. S. (2007). PPAR*γ* regulates adipose triglyceride lipase in adipocytes in vitro and in vivo. *American Journal of Physiology-Endocrinology and Metabolism*.

[35] Naderali M. M., Itua I., Abubakari A. R., Naderali E. K. (2012). Short-Term therapy with rosiglitazone, a PPAR-gamma Agonist, improves metabolic profile and vascular function in nonobese lean wistar rats. *ISRN Pharmacol*.

[36] Harrington W. W., Britt C. S., Wilson J. G. (2007). The effect of PPAR*α*, PPAR*δ*, PPAR*γ*, and PPARpan agonists on body weight, body mass, and serum lipid profiles in diet-induced obese AKR/J mice. *PPAR Research*.

[37] Laplante M., Festuccia W. T., Soucy G. (2009). Tissue-specific postprandial clearance is the major determinant of PPARgamma-induced triglyceride lowering in the rat. *American Journal of Physiology-Regulatory, Integrative and Comparative Physiology*.

[38] Blanchard P.-G., Festuccia W. T., Houde V. P. (2012). Major involvement of mTOR in the PPARgamma-induced stimulation of adipose tissue lipid uptake and fat accretion. *Journal of Lipid Research*.

[39] Auwerx J., Schoonjans K., Fruchart J.-C., Staels B. (1996). Transcriptional control of triglyceride metabolism: Fibrates and fatty acids change the expression of the LPL and apo C-III genes by activating the nuclear receptor PPAR. *Atherosclerosis*.

[40] Sheikh K., Camejo G., Lanne B., Halvarsson T., Landergren M. R., Oakes N. D. (2007). Beyond lipids, pharmacological PPARalpha activation has important effects on amino acid metabolism as studied in the rat. *American Journal of Physiology-Endocrinology and Metabolism*.

[41] Vigerust N. F., Cacabelos D., Burri L. (2012). Fish oil and 3-thia fatty acid have additive effects on lipid metabolism but antagonistic effects on oxidative damage when fed to rats for 50 weeks. *The Journal of Nutritional Biochemistry*.

[42] Ribel-Madsen A., Ribel-Madsen R., Brøns C., Newgard C. B., Vaag A. A., Hellgren L. I. (2016). Plasma acylcarnitine profiling indicates increased fatty acid oxidation relative to tricarboxylic acid cycle capacity in young, healthy low birth weight men. *Physiological Reports*.

[43] Ueland T., Svardal A., Øie E. (2013). Disturbed carnitine regulation in chronic heart failure - Increased plasma levels of palmitoyl-carnitine are associated with poor prognosis. *International Journal of Cardiology*.

[44] Yang L., Zhang Y., Wang S., Zhang W., Shi R. (2014). Decreased liver peroxisomal *β*-oxidation accompanied by changes in brain fatty acid composition in aged rats. *Neurological Sciences*.

[45] Baranowski M., Blachnio-Zabielska A., Gorski J. (2009). Peroxisome proliferator-activated receptor alpha activation induces unfavourable changes in fatty acid composition of myocardial phospholipids. *Journal of Physiology and Pharmacology*.

[46] DeLany J. P., Windhauser M. M., Champagne C. M., Bray G. A. (2000). Differential oxidation of individual dietary fatty acids in humans. *American Journal of Clinical Nutrition*.

[47] Ichi I., Kono N., Arita Y. (2014). Identification of genes and pathways involved in the synthesis of Mead acid (20:3n - 9), an indicator of essential fatty acid deficiency. *Biochimica et Biophysica Acta (BBA) - Molecular and Cell Biology of Lipids*.

[48] Arab L. (2003). Biomarkers of Fat and Fatty Acid Intake. *Journal of Nutrition*.

[49] Flanagan J. L., Simmons P. A., Vehige J., Willcox M. D., Garrett Q. (2010). Role of carnitine in disease. *Journal of Nutrition and Metabolism*.

