# Supplementary material for: Short-Term Activation of Peroxisome Proliferator-Activated Receptors α and γ Induces Tissue-Specific Effects on Lipid Metabolism and Fatty Acid Composition in Male Wistar Rats
Source: PPAR Res. 2019 Jun 12;2019:8047627. doi: 10.1155/2019/8047627 (PMC6594300; doi:10.1155/2019/8047627)
Supplement: Supplementary Materials — Supplemental Figure 1: individual data on weights and lipid related parameters in Wistar rats during treatment with PPAR agonists for 12 days. Supplemental Figure 2: individual data on hepatic fatty acid composition (wt%) in Wistar rats after treatment with PPAR agonists for 12 days. Supplemental Figure 3: plasma fatty acid composition (wt%) illustrated as (A) lines indicating geometric mean (gSD) values and as (B) individual raw data on plasma fatty acid composition (wt%) in Wistar rats after treatment with PPAR agonists for 12 days. Red bars correspond to PPARα vs control and blue bars to PPARγ vs control. ANOVA, analysis of variance; MUFA, monounsaturated fatty acids; PPAR, peroxisome proliferator-activated receptor; PUFA, polyunsaturated fatty acids; SFA, saturated fatty acids. Supplemental Figure 4: individual data on cardiac fatty acid composition (wt%) in Wistar rats after treatment with PPAR agonists for 12 days. Supplemental Figure 5: individual hepatic gene expression, normalized towards the control group. Supplemental Figure 6: individual epididymal adipose tissue gene expression, normalized towards the control group. [file 8047627.f1.docx]

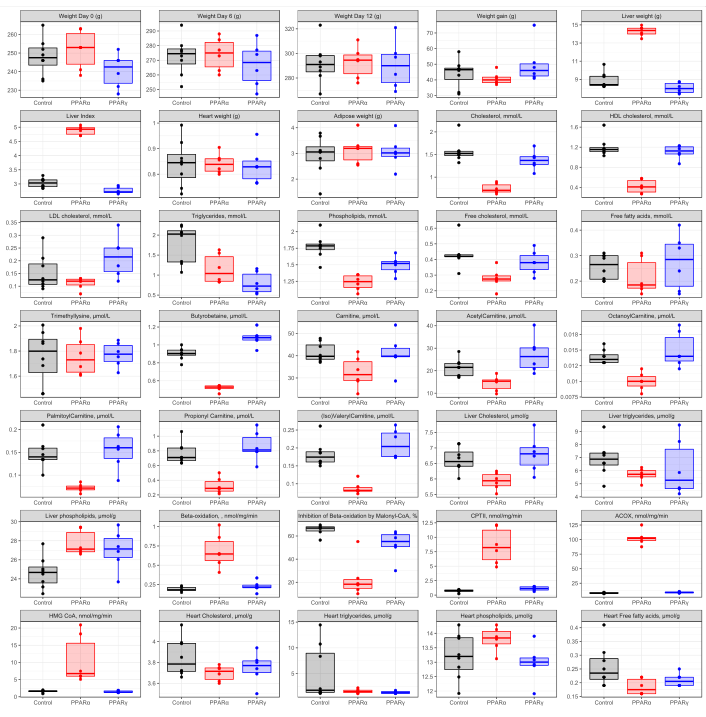


**Supplemental figure 1.** Individual data on weights and lipid related parameters in Wistar rats during treatment with PPAR agonists for 12 days.


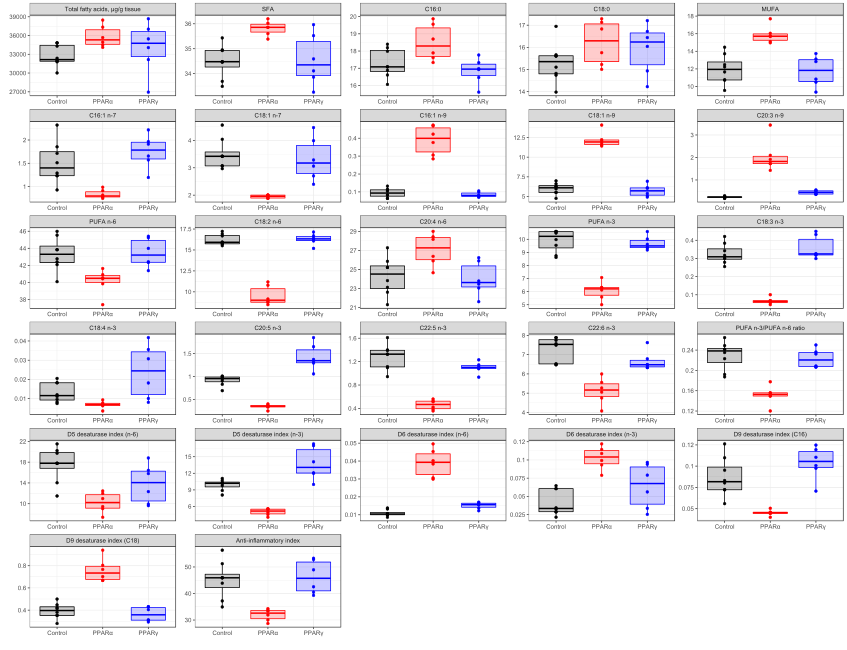


## **Supplemental figure 2.** Individual data on hepatic fatty acid composition (wt%) in Wistar rats after treatment with PPAR agonists for 12 days.


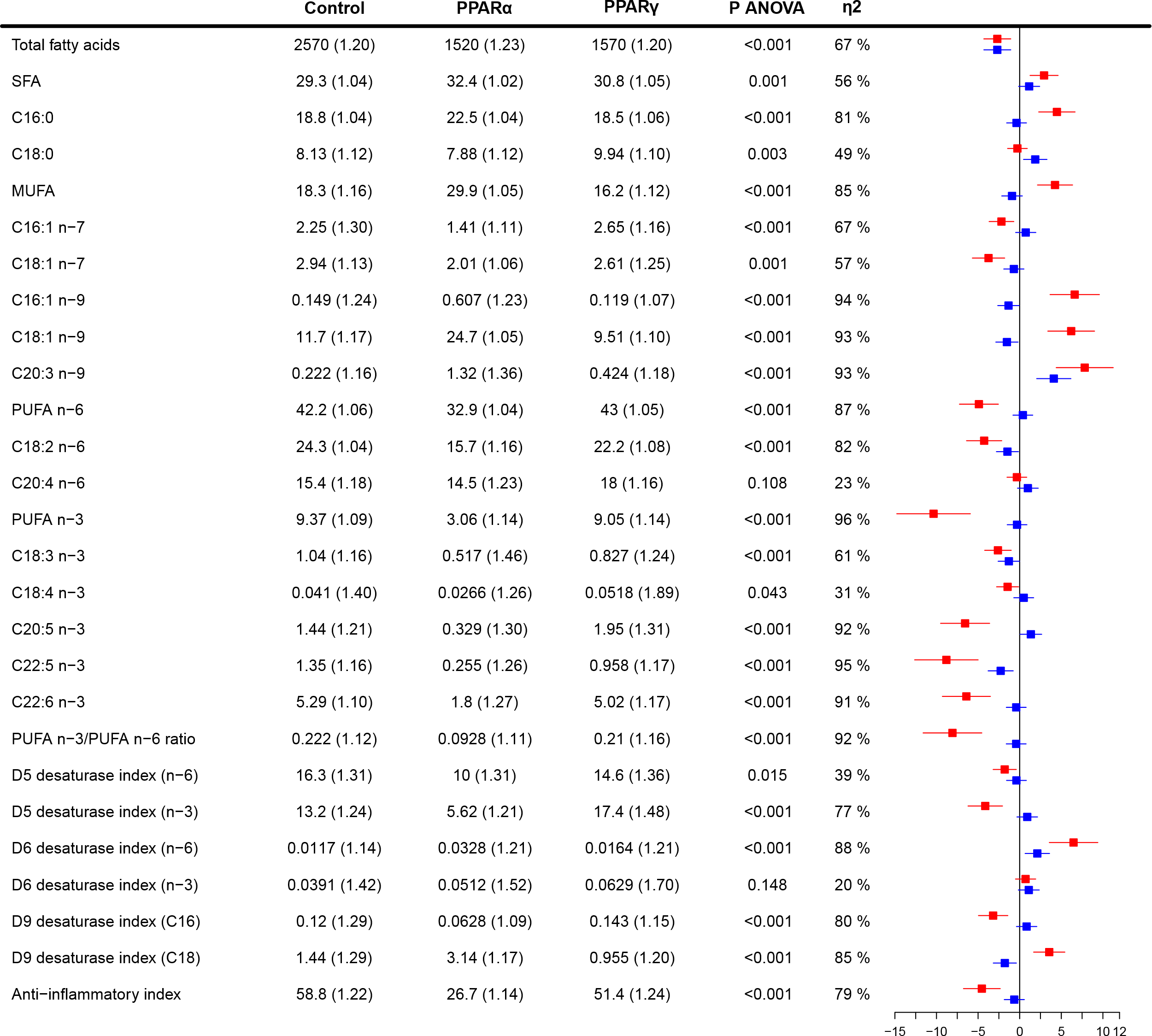


**A**


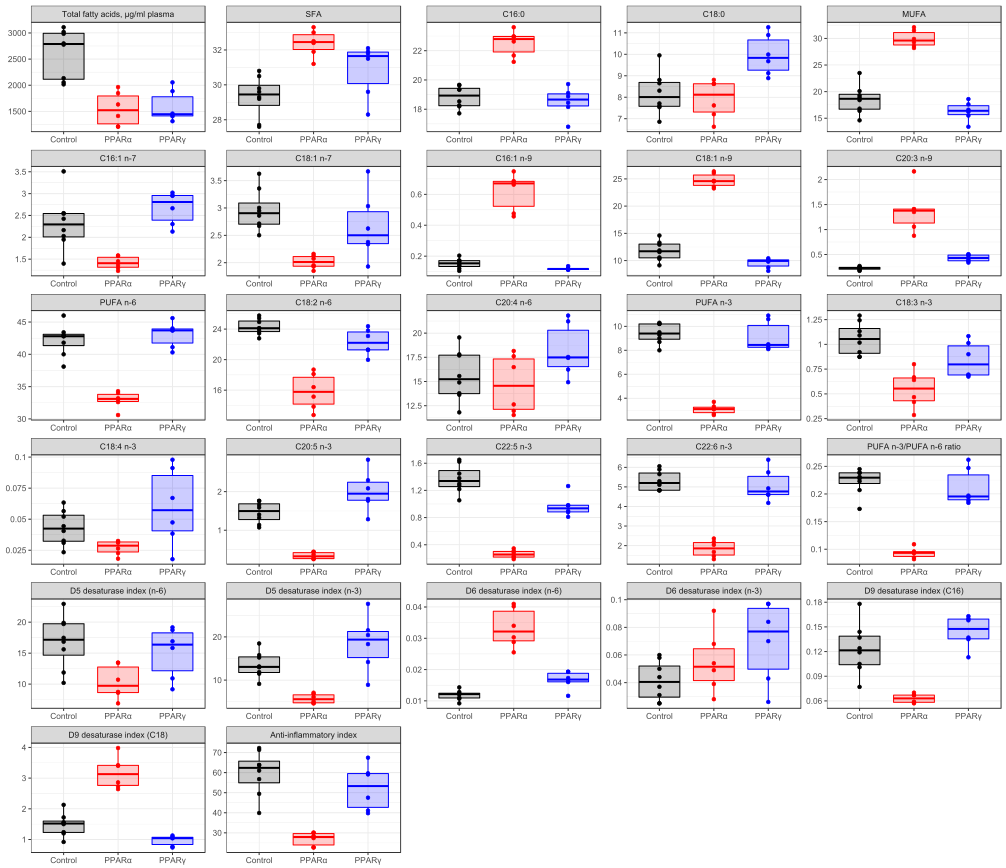


**B**

| **Supplemental figure 3.** Plasma fatty acid composition (wt%) illustrated as (**A**) lines indicating geometric mean (gSD) values and as (**B**) individual raw data on plasma fatty acid composition (wt%) in Wistar rats after treatment with PPAR agonists for 12 days. Red bars correspond to PPARα vs control, blue bars to PPARγ vs control. Abbreviations: ANOVA, analysis of variance; MUFA, monounsaturated fatty acids; PPAR, peroxisome proliferator-activated receptor; PUFA, polyunsaturated fatty acids; SFA, saturated fatty acids. |  |
| --- | --- |
| D5 desaturase index (n-6) = C20:4n-6 / C20:3n-6 (an indirect index of Δ5 desaturase activity based on n-6 PUFA) | |
| D5 desaturase index (n-3) = C20:5n-3 / C20:4n-3 (an indirect index of Δ5 desaturase activity based on n-3 PUFA) | |
| D6 desaturase index (n-6) = C18:3n-6 / C18:2n-6 (an indirect index of Δ6 desaturase activity based on n-6 PUFA) | |
| D6 desaturase index (n-3) = C18:4n-3 / C18:3n-3 (an indirect index of Δ6 desaturase activity based on n-3 PUFA) | |
| D9 desaturase index (C16) = C16:1n-7 / C16:0 (an indirect index of Δ9 desaturase activity based on C16 SFA/MUFA) | |
| D9 desaturase index (C18) = C18:1n-9 / C18:0 (an indirect index of Δ9 desaturase activity based on C18 SFA/MUFA) | |
| Anti-inflammatory index = ((C22:6n-3 + C22:5n-3 + C20:3n-6 + C20:5n-3) / C20:4n-6)*100 |  |


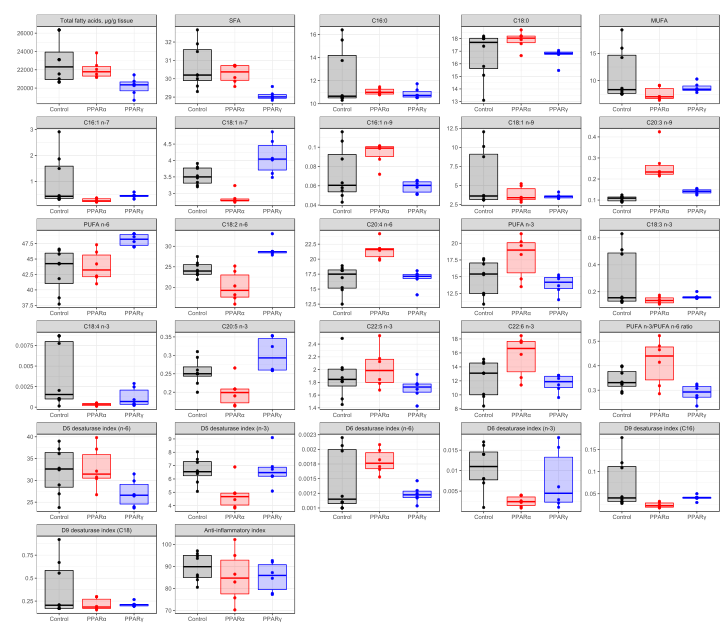


## **Supplemental figure 4.** Individual data on cardiac fatty acid composition (wt%) in Wistar rats after treatment with PPAR agonists for 12 days.


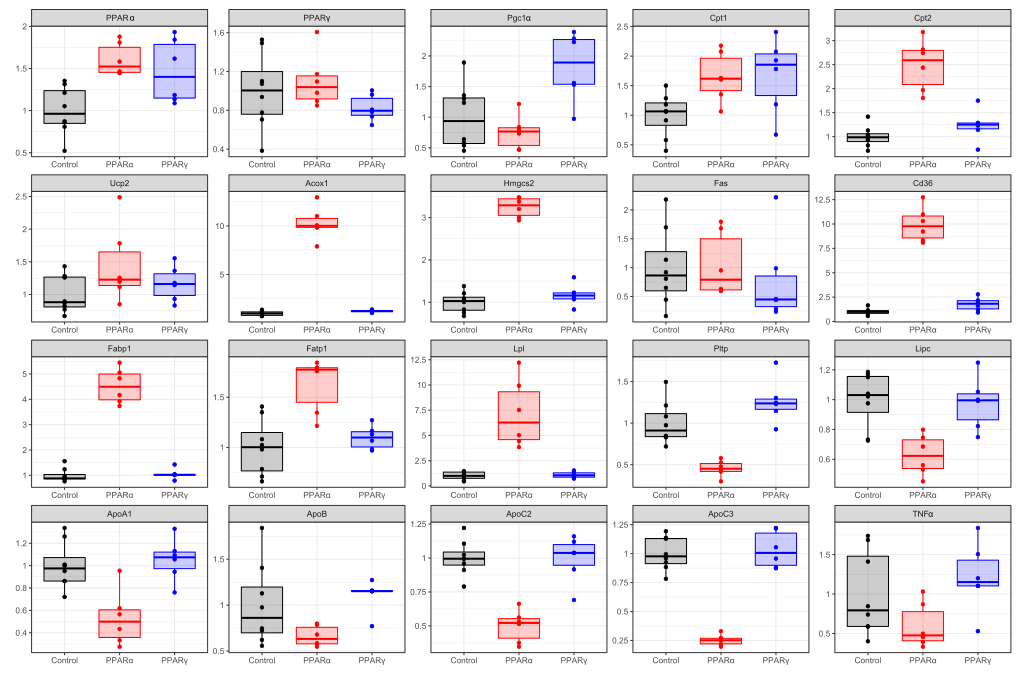


## **Supplemental figure 5.** Individual hepatic gene expression, normalized towards the control group


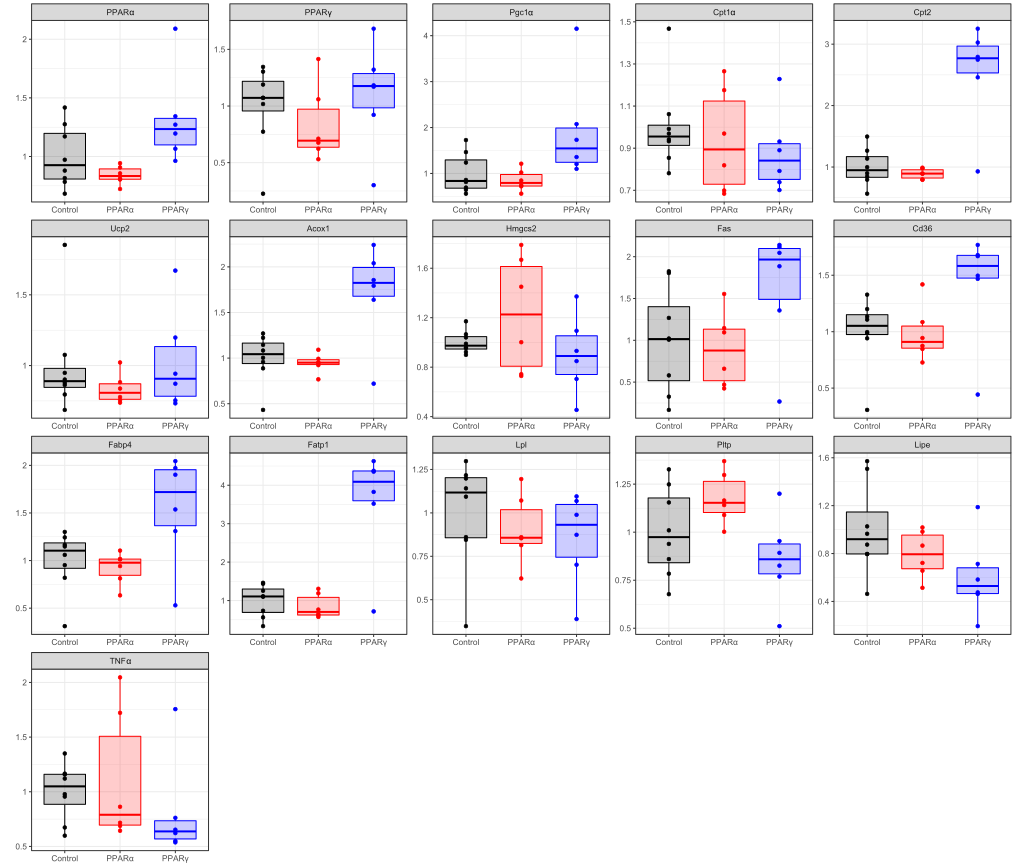


**Supplemental figure 6.** Individual epididymal adipose tissue gene expression, normalized towards the control group.
